# M6A Demethylase ALKBH5 in Human Diseases: From Structure to Mechanisms

**DOI:** 10.3390/biom15020157

**Published:** 2025-01-21

**Authors:** Miaochun Fang, Liwen Ye, Yue Zhu, Linying Huang, Shun Xu

**Affiliations:** Guangdong Provincial Key Laboratory of Medical Immunology and Molecular Diagnostics, Institute of Aging Research, School of Medical Technology, Guangdong Medical University, Songshan Lake, Dongguan 523808, China; fangmiaochun@gdmu.edu.cn (M.F.); yeliwen@gdmu.edu.cn (L.Y.); zy12581@gdmu.edu.cn (Y.Z.); huanglinying@gdmu.edu.cn (L.H.)

**Keywords:** N6-methyladenosine, biological function, inhibitor

## Abstract

N6-methyladenosine (m6A) is the most abundant, dynamically reversible, and evolutionarily conserved internal chemical modification in eukaryotic RNA. It is emerging as critical for regulating gene expression at the post-transcriptional level by affecting RNA metabolism through, for example, pre-mRNA processing, mRNA decay, and translation. ALKBH5 has recently been identified as an endogenous m6A demethylase implicated in a multitude of biological processes. This review provides an overview of the structural and functional characteristics of ALKBH5 and the involvement of ALKBH5 in diverse human diseases, including metabolic, immune, reproductive, and nervous system disorders, as well as the development of inhibitors. In summation, this review highlights the current understanding of the structure, functions, and detailed mechanisms of ALKBH5 in various physiological and pathological processes and provides valuable insights for clinical applications and foundational research within related fields.

## 1. Introduction

N6-methyladenosine (m6A) is the most prevalent, rich, and highly conserved internal modification in eukaryotic RNA [1], which is enriched in the long internal exon, stop codon, or 3ʹ untranslated region (3ʹ UTR) of messenger RNA (mRNA) and long noncoding RNAs (lncRNAs). It usually occurs in the consensus motif of RRACH (R = G or A; H = A, C, or U). m6A modification has emerged as a post-transcriptional regulator of gene expression [2], which plays a pivotal role in RNA function and processing, including in maintaining RNA stability, regulating RNA splicing, and translation [3]. An increasing number of studies have demonstrated that m6A methylation is implicated in multiple biological processes and is strongly associated with the occurrence and development of various human diseases [4,5], including azoospermia [6], cancer [7,8], type 2 diabetes mellitus (T2DM) [9,10], and metabolic dysfunction-associated steatotic liver disease (MASLD) [11]. Hence, exploring the potential functions and detailed mechanisms of m6A methylation in human diseases can deepen the understanding of development and pathology and, based on novel insights, provide a strategy for diagnosing and therapeutically treating human diseases.

The abundance and effect of m6A methylation on RNA are regulated by dynamic interactions among methyltransferases (“writers”), demethylases (“erasers”), and binding proteins (“readers”) [2,12]. AlkB homolog 5 (ALKBH5) is one of the two major identified endogenous m6A demethylases [13] and belongs to the AlkB subfamily of Fe (II)/αKG dioxygenases [14]. ALKBH5 influences gene expression by mediating m6A demethylation, affecting multiple events in RNA metabolism, including pre-mRNA processing, mRNA decay, and translation [15], thus participating in multiple physiological and pathological processes [4,15]. In this review, we present a comprehensive overview of research advances regarding ALKBH5, focusing on its structure, biological role, and potential mechanisms in various human diseases. In addition, this review highlights the development of novel ALKBH5 inhibitors and reveals their applications.

## 2. Structural Features and Catalytic Mechanisms of ALKBH5

**Figure 1:**
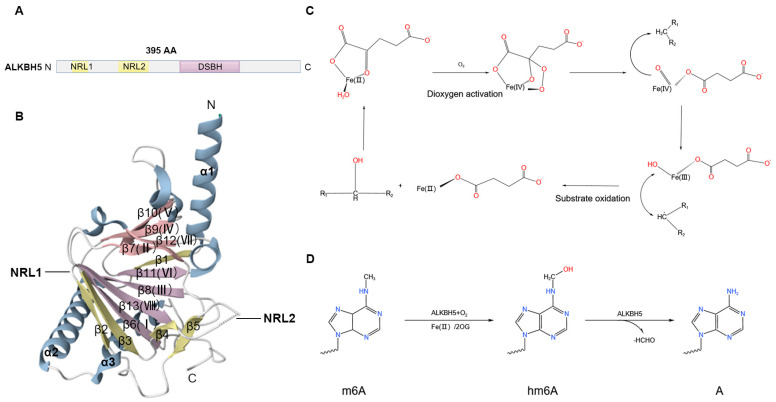
Schematic diagram of the structure and catalytic mechanism of ALKBH5. (**A**) The human *ALKBH5* gene encodes a protein containing 395 amino acids. (**B**) The three-dimensional spiral structure of ALKBH5. Three α helices are depicted in blue, the major β-sheet in purple, and the minor β-sheet in pink, and NRL1 and NRL2 are indicated (PDB ID: 4NJ4 [16]). (**C**) The demethylation mechanism of general oxygenases. Two steps, namely, the activation of dioxygen and the oxidation of the substrate, are implicated in the oxidation reaction. (**D**) Demethylation mechanism of ALKBH5 to m6A. M6A is oxidized by ALKBH5 to hydroxymethyl-A intermediates and then formaldehyde is removed from hydroxymethyl-A to obtain adenine ((**C**,**D**) the chemical formula was created using the KingDraw app version 3.0.2.20).

ALKBH5 belongs to the AlkB subfamily, which is the first group of 2OG oxygenases characterized as methylated nucleic acid N-demethylases [16] and comprises nine members, including ALKBH 1-8 and fat mass and obesity-associated protein (FTO) [17]. The human *ALKBH5* gene is localized in the 17p11.2 region and encodes a protein containing 395 amino acid residues with a molecular weight of approximately 43 kD [18] (Figure 1A), which catalyzes the oxidation of a wide range of substrates, including nucleic acids, lipids, proteins, and small-molecule metabolites [19]. The core structure of ALKBH5 consists of a double-stranded β-helix (DSBH) domain and two nucleotide recognition loops (NRL1 and NRL2) (Figure 1A), which are characteristic features of the 2OG dioxygenase AlkB family [17].

The core DSBH fold of ALKBH5 contains eight antiparallel β-strands (βI-VIII), which form two β-sheets: the major β-sheet (strands β6, 8, 11, and 13) and the minor β-sheet (strands β7, 9, 10, and 12) (Figure 1B). Moreover, three extra β-strands (β1, β2, and β3) extend the major β-sheet, and three helices (α1, α2, and α3) flank the DSBH [20] (Figure 1B). The DSBH domain determines the demethylase activity of ALKBH5 and indirectly influences the additional functional domains involved in demethylation [21]. The DSBH serves as a scaffold for the three Fe (II)-binding residues (His204, Asp206, and His266), which constitute a conserved HXDXnH module for coordinating metal ions [16]. The 2OG (2-oxoglutarate) binding pocket is located in the cavity between the two β-sheets of DSBH and provides the substrate access to the active site [15,20]. In the presence of the substrate, the βIV–V loop and NRL2 are folded to enclose the substrate at the active site. The active site, a catalytically inert Mn ion (substituted for catalytically active Fe (II)), is coordinated by the highly conserved metal binding triplets (His204, Asp206, and His266), water molecules, and 2OG, NOG (N-oxalylglycine), or sulfate ions [19]. Normally, Fe (II) has catalytic activity and plays a key role in catalytic reactions. It participates in processes like substrate activation and electron transfer. However, when it is replaced by Mn ions, it is possible that the biochemical reaction processes of ALKBH5, such as substrate modification, cannot proceed normally or that the efficiency of these processes may be reduced. In short, the active site plays a role in the related complexes based on its specific structure, coordination, and binding between components. ALKBH5 has a shorter NRL1 than the other ferrous iron-dependent nucleic acid oxygenases (ALKBH1, ALKBH2, ALKBH3, and FTO) [19]. It lies in the N-terminal extension of ALKBH5, contains β-strands 2 and 3 [19,22], extends the major β-fold of DSBH, and forms a short type-I β-turn. NRL2 is disordered in the apical portion and is sandwiched between DSBH strand βII and the C-terminal, which consists of β-strands 4 and 5. The sequence of the disordered apex of ALKBH5 NRL2 contains two basic residues, Lys147 and Arg148, which play important roles in substrate recognition by interacting with the phosphate backbone of the RNA substrate [16].

It has been reported that the demethylation mechanism of Fe (II)- and 2OG-dependent oxygenase involves two oxidation reaction steps: dioxygen activation and substrate oxidation (Figure 1C). Initially, Fe (II) and 2OG each contribute two electrons to activate the dioxygen molecule. The activated dioxygen molecule transitions to a bridging peroxide and then to a Fe (IV)-oxo intermediate. In the substrate oxidation stage, the inert C-H bonds of RNA or other substrates are oxidized to a hydroxyl group by the highly active Fe (IV)-oxo species, and one formaldehyde molecule is removed from this intermediate to yield the final demethylation product. Simultaneously, Fe (IV) is reduced back to Fe (II) to complete the catalytic cycle, reducing 2OG to succinate (Figure 1C). Due to the instability of C-N bonds, N-methylated substrates undergo hydrolytic deformylation, triggering direct demethylation [18,23]. Similarly, ALKBH5 demethylates m6A in mRNA by oxidizing N6-methyl, forming a transient N6-hydroxymethyladenosine (hm6A) intermediate [19] (Figure 1D). This modification is highly unstable and spontaneously decomposes into adenosine within a few hours [24]. The removal of one formaldehyde molecule from this intermediate yields the final demethylation product [22], along with succinate, formaldehyde, and carbon dioxide [23]. ALKBH5 directly converts m6A to adenosine (A), rapidly releasing formaldehyde (FA), and thus, only demethylated adenosine products are observed under ALKBH5 catalysis [22] (Figure 1D).

## 3. Biological Functions of ALKBH5

**Figure 2:**
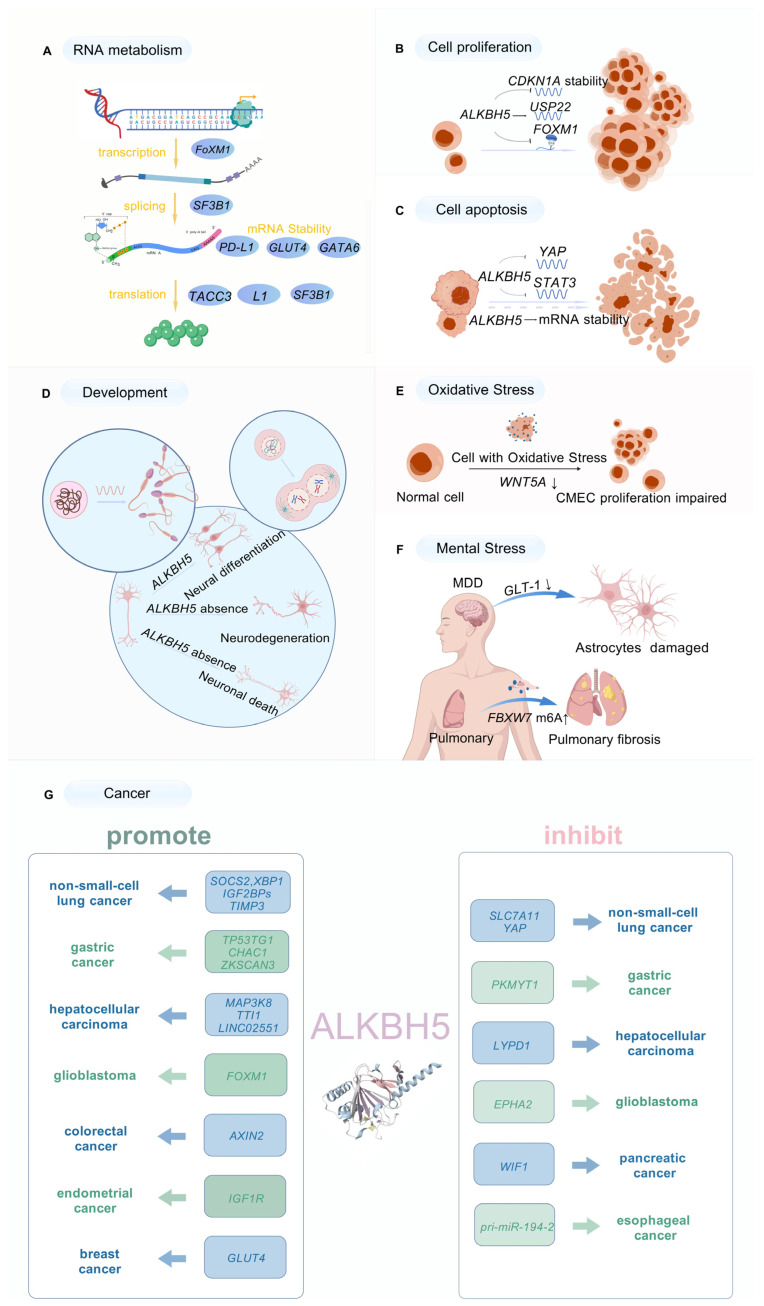
Biological functions of ALKBH5. (**A**) ALKBH5 regulates RNA metabolism by participating in RNA splicing, translation, and mRNA stability. (**B**) ALKBH5 mediates cell proliferation. Dashed lines indicate inhibition, and solid lines indicate facilitation. (**C**) ALKBH5 is associated with apoptosis. Dashed lines indicate inhibition, and solid lines indicate facilitation. (**D**) ALKBH5 is involved in development. ALKBH5 is essential for maintaining normal sperm development and oocyte meiosis, and also regulates neuronal development, including neuronal cell differentiation, axon growth, and synapse formation. (**E**) ALKBH5 regulates oxidative stress. (**F**) ALKBH5 plays an important role in mental stress. (**G**) The impact of ALKBH5 in cancer. The bidirectional regulatory role of ALKBH5 in different cancers; that is, it can promote the development of certain cancers and also inhibit the development of some other cancers. (Created with BioGDP.com; the agreement number is GDP2024ZWB7HW).

*ALKBH5* is predominantly localized in the Golgi apparatus, cytosol, and nuclear speckles [25], where it participates in multiple biological processes, including RNA metabolism, cell proliferation, apoptosis, development, stress response, and cancer, via mediating the m6A demethylation of various substrates (Figure 2).

### 3.1. Roles of ALKBH5 in RNA Metabolism

*ALKBH5* is localized in nuclear speckles, which are associated with mRNA splicing factors, indicating a functional link between m6A and mRNA splicing [26] (Figure 2A). During spermatogenesis, in pachytene spermatocytes and round and elongated spermatids, *ALKBH5* participates in regulating RNA splicing to ensure the normal processing of long 3’UTR mRNAs, while the absence of *ALKBH5* results in abnormal splicing and shorter transcripts, leading to male sterility [27]. Moreover, *ALKBH5* can indirectly influence mRNA splicing through the regulation of key splicing factors. *SF3B1* is the most frequently mutated splicing factor in myelodysplastic syndrome (MDS)—a clonal hematopoietic disorder with a variable risk of leukemic transformation [28]. *ALKBH5* drives 5’UTR m6A demethylation and fine-tunes *SF3B1* translation, which directs the splicing of central DNA repair and epigenetic regulators during transformation (a process in which recipient bacteria directly ingest free DNA fragments from donor bacteria to acquire new genetic traits), affecting genomic stability and leukemia progression [29]. Kwangseog Ahn et al. showed that *ALKBH5* inhibits L1 retrotransposons, thus reducing the efficiency of translation [30] (Figure 2A). Increased levels of poly(A) mRNA were observed in the nuclei of *ALKBH5* knockout cells [25], suggesting that *ALKBH5* affected the assembly of mRNA processing factors and possibly undermined the efficiency of mRNA export from the nucleus.

*ALKBH5* may play dual roles in regulating RNA stability (Figure 2A). Pei et al. reported that the absence of *ALKBH5* disrupted the stability of *GATA6* mRNA [31]. Chen et al. also proved that *ALKBH5* knockout accelerated *PD-L1* mRNA degradation, while the overexpression of *ALKBH5* significantly enhanced the stability of *PD-L1* mRNA [32]. Similarly, Liu et al. demonstrated that *ALKBH5* upregulation promoted m6A demethylation, increasing the stability and expression of *GLUT4* mRNA [33]. These studies suggest that *ALKBH5* plays a positive role in maintaining the stability and function of target mRNAs. In contrast, *ALKBH5* is tightly associated with mRNA degradation. Zhan et al. reported that *ALKBH5* destabilized *PHF20* mRNA by reducing its methylation, thus suppressing colorectal cancer (CRC) [34]. The overexpression of *ALKBH5* in GC-2 cells significantly diminished the stability and expression levels of *PLOD2* mRNA [35]. Hence, the role of *ALKBH5* in RNA stability is still ambiguous and far from being elucidated.

In addition to the post-transcriptional regulation of target genes, *ALKBH5* can indirectly affect RNA transcription by modulating the expression of various transcription factors (Figure 2A). A study on acute myeloid leukemia (AML) has shown that *ALKBH5* regulated the expression of *TACC3* (a transcription factor) in an m6A-dependent manner, which critically influenced leukemic cell transformation and AML development [36]. Transcription factor *FOXM1* is a key cell cycle molecule required for G1/S and G2/M transitions and M phase progression [37]. Research by Huang et al. revealed that *ALKBH5* deficiency reduced the nascent transcripts of *FOXM1* in glioblastoma stem-like cells, subsequently resulting in detectable changes in mature RNA [38]. These results show that *ALKBH5* indirectly affects the transcriptional regulatory network through the demethylation of transcription factors.

### 3.2. ALKBH5 Mediation of Cell Proliferation

An increasing number of studies have unveiled that the silencing of *ALKBH5* delays the progression of the cell cycle by arresting cells in the G0/G1 phase and inhibiting cell proliferation (Figure 2B). For instance, in *ALKBH5* knockdown cells, the m6A level and stability of *CDKN1A* mRNA are upregulated, which enhances the expression of CDKN1A and suppresses the proliferation of esophageal squamous cell carcinoma (ESCC) [39]. Additionally, in glioblastoma stem-like cells (GSCs), *ALKBH5*-demethylated *FOXM1* nascent transcripts promote the proliferation of GSCs [38]. Furthermore, ALKBH5-mediated m6A deficiency increases the expression of USP22 and RNF40 in osteosarcomas, promoting osteosarcoma cell growth and proliferation [40]. What is more, research has discovered that ALKBH5 was highly expressed in AML cells and that *ALKBH5* knockdown diminished the clonogenic ability of AML cells, indicating that ALKBH5 promoted the proliferation of leukemia cells [36,41].

### 3.3. Association of ALKBH5 with Apoptosis

ALKBH5 plays a complex role in regulating apoptosis (Figure 2C). In osteosarcoma cells, the overexpression of *ALKBH5* inhibits the m6A methylation of *pre-miR-181b-1 and YAP*-mRNA, which significantly triggers apoptosis [42], suggesting that ALKBH5 accelerates apoptosis. In osteosarcoma cells, the enhanced expression of ALKBH5 weakens the stability of *SOCS3* mRNA in an m6A-dependent manner, inactivating the *STAT3* signaling pathway and increasing cell apoptosis [43]. Alternatively, *ALKBH5* knockdown can significantly increase the proportion of apoptotic AML cells and leukemia stem cells [41], suggesting that *ALKBH5* silencing also promotes cell apoptosis. *Alkbh5* deficiency enhanced the m6A level of mRNA in male mouse testicular tissue, thus altering the expression of 18 mRNAs related to spermatogenesis, which promoted the apoptotic rate of meiotic metaphase-stage spermatocytes [25].

### 3.4. Involvement of ALKBH5 in Development

ALKBH5 exerts a regulatory effect on sperm and oocyte development (Figure 2D). ALKBH5 has been demonstrated to exhibit a higher expression level in mouse testes than in other tissues [38,44]. The ablation of ALKBH5 in mice impeded sperm formation and reduced sperm quantity, ultimately leading to male infertility [25]. Further research revealed that *ALKBH5* ensured the normal processing of related mRNA during spermatogenesis by regulating the cleavage and stability of long 3’UTR mRNA. The absence of *ALKBH5* led to spermatocyte apoptosis and abnormal spermatogenesis during meiosis [27], suggesting that ALKBH5 played an essential role in maintaining normal sperm development and male mouse fertility. ALKBH5 has also been reported to play a potential role in regulating ovarian function and endometrium physiology. For example, the upregulation of m6A mediated by *ALKBH5* deletion hindered the timely attenuation of RNA during oocyte meiosis, which resulted in widespread defects in oocytes and led to female infertility [45].

In addition, ALKBH5 plays a regulatory role in neuronal developmental processes such as neuronal cell differentiation, neuron axon growth, and synapse formation. Under low pressure and oxygen conditions, ALKBH5 deficiency disrupted the m6A mRNA methylation balance in the mouse cerebellum and then markedly accelerated mRNA nuclear export, which altered the phenotypes in the cerebellum, including neuronal structural disorder, abnormal cell proliferation and differentiation, and slow cerebellar development [46]. Another study proved that Alkbh5 knockdown exacerbated neuronal damage [47].

### 3.5. ALKBH5 Regulation of Oxidative Stress Response

Recently, a study revealed that *ALKBH5*-mediated alteration in the methylation status of mRNA in cells is associated with the cellular response under oxidative stress (Figure 2E). Specifically, *ALKBH5* was significantly upregulated when exposed to oxidative stress, which led to an overall decrease in m6A and reduced the expression of *WNT5A* via post-transcriptional mRNA modulation, thereby impairing proliferation, migration, and tube formation in hypoxic microvascular endothelial cells (CMECs) and ultimately affecting the cellular response to oxidative stress [48]. Moreover, the overproduced mitochondrial reactive oxygen species (mtROS) in alveolar epithelial cells during 1-NP-induced pulmonary fibrosis enhanced the *ALKBH5* SUMOylation modification and led to an increased m6A level of *FBXW7* mRNA, which was integral for TRF2 degradation and in cellular senescence [49]. These studies indicate that ALKBH5 can affect cellular responsiveness to oxidative stress by mediating changes in mRNA methylation, ultimately affecting cellular status.

### 3.6. ALKBH5 Regulation of Mental Stress Response

ALKBH5 has a non-negligible impact on mental stress (Figure 2F). Research has revealed ALKBH5 hyperactivation in patients with major depressive disorder (MDD) and in a depressive mouse model [50]. *ALKBH5* diminished the m6A level of glutamate transporter-1 (*GLT-1*) and increased its expression in mouse brain astrocytes, which improved the morphological atrophy and functional neuronal deficits caused by chronic stress [51]. These studies indicate that ALKBH5 plays an important role in mental stress responses, providing novel insights into the identification of potential therapeutic targets for mental-stress-related diseases.

### 3.7. Impact of ALKBH5 on Cancer

Accumulating evidence has demonstrated that ALKBH5 plays a critical role in tumorigenesis and tumor development, impacting tumor initiation, progression, and metastasis by regulating the mRNA metabolism of oncogenic and tumor suppressor transcripts [52] (Figure 2G). ALKBH5 was highly expressed in various cancers, including non-small-cell lung cancer [53,54,55], glioblastoma (GBM) [38], hepatocellular carcinoma [56], colorectal cancer [57], gastric cancer [58], endometrial cancer [59], and breast cancer [60], with high expression of ALKBH5 being closely related to the malignancy of these tumors. In contrast, a series of studies implicated ALKBH5 as a tumor suppressor in diverse caners, including non-small-cell lung cancer [61], esophageal cancer [62], pancreatic cancer [63], gastric cancer, and so on. Obviously, understanding the impact of ALKBH5 on tumorigenesis is highly significant for exploring the pathogenesis and clinical treatment of tumors. However, the function and underlying mechanisms of ALKBH5 in tumor development are still ambiguous and require further investigations.

## 4. Research on ALKBH5 in Human Diseases

### 4.1. Association of ALKBH5 with Metabolic Disorders

**Figure 3:**
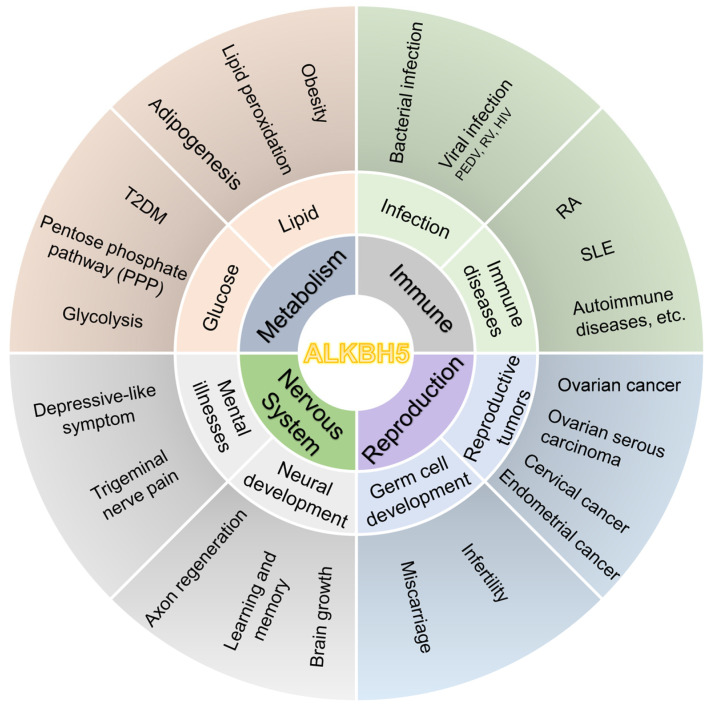
ALKBH5 is involved in various diseases. ALKBH5 exerts critical roles in glucolipid metabolism, bacterial and viral infection, RA, SLE, reproductive system cancers, infertility, and miscarriage. In addition, ALKBH5 takes part in neurological disorders, such as brain damage and depression. (This picture was created with PowerPoint version 16051.18227.20162.0.)

A growing body of recent studies has focused on the relationship between ALKBH5 and metabolic diseases, revealing that ALKBH5 plays a major role in glucolipid metabolism and metabolic disorders by regulating relevant genes and signaling pathways (Figure 3).

#### 4.1.1. ALKBH5 and Glucose Metabolism

ALKBH5 influences glucose metabolism through regulating the expression of glucose metabolism-associated genes [64] in glycolysis, aerobic oxidation, the pentose phosphate pathway, glycogen synthesis, and gluconeogenesis [65] (Table 1). Initially, ALKBH5 exerts a dual influence on the glycolysis pathway via the regulation of the m6A demethylation of related genes. One research team has reported that ALKBH5 exerted an inhibitory effect on bladder cancer by disrupting the glycolytic process in bladder cancer cells [66]. Mechanistically, ALKBH5 impeded the progression of bladder cancer and enhanced the sensitivity of bladder cancer cells to cisplatin by modulating the glycolysis pathway through CK2α in an m6A-dependent manner [66]. *ALKBH5* stabilized *FLII* mRNA in an m6A-YTHDF2-dependent manner, thus suppressing glycolysis, cell proliferation, invasion, and PARD progression [67]. Moreover, in a high-fat environment, downregulated *FTO* and *ALKBH5* cooperatively activated FOXO signaling through IGF2BP2-mediated m6A methylation in *HK2* mRNA, which boosted glycolysis in colorectal cancer [68]. In contrast, the increased expression of *ALKBH5* promoted m6A demethylation and the stability of *GLUT4* mRNA in a YTHDF2-dependent manner, which resulted in enhanced glycolysis in drug-resistant breast cancer cells [33].

Furthermore, Han et al. have reported that *PRMT6* directly methylated ALKBH5 at Arg283, and the methylated ALKBH5 strengthened the stability of *LDHA* mRNA, leading to increased aerobic glycolysis in breast cancer cells [60], which suggested that ALKBH5 might be involved in regulating aerobic glycolysis. In addition, upregulated *ALKBH5* demethylated *G6PD* mRNA and enhanced the stability and expression of G6PD in glioma, which activated the pentose phosphate pathway and stimulated the proliferation of glioma cells [69].

#### 4.1.2. ALKBH5 and Lipid Metabolism

*ALKBH5* is known to regulate adipogenesis by altering the m6A modification of mRNA in lipid-related genes (Table 2). The downregulated expression of *ALKBH5* reinforced the m6A methylation of *LCAT* to improve the stability of its mRNA, which promoted preadipocyte differentiation and thus enhanced adipogenesis in chickens [72], suggesting that ALKBH5 might be a checkpoint for determining preadipocyte fate. Consistently, ALKBH5 negatively regulated adipogenesis in mesenchymal stem cells (MSCs) [73]. The diminished expression of *ALKBH5* enhanced *TRAF4* m6A modification, thus reducing the expression of TRAF4, and the PKM2/TRAF4 interaction, which weakened the kinase activity of PKM2 and obstructed β-catenin signal transduction, thus promoting fat formation in MSCs [73]. However, Chen et al. reported that curcumin treatment decreased the expression of *ALKHB5*, which caused a higher m6A level in *TRAF4* mRNA that was recognized by and combined with YTHDF1 to promote *TRAF4* translation. The enhanced expression of TRAF4 facilitated the degradation of PPARγ through the ubiquitin–proteasome pathway, thus inhibiting adipogenesis [74], which indicated that ALKBH5 might inhibit lipogenesis by regulating the degradation of lipid differentiation factors in an m6A-dependent manner.

Additionally, ALKBH5 affects lipid metabolism by regulating classical signaling pathways. The overexpression of ALKBH5 significantly increased FABP5 expression in an m6A-IGF2BP2-dependent manner, activating the PI3K/Akt/mTOR signaling pathway and enhancing lipid metabolism in pancreatic neuroendocrine neoplasms (pNENs) [75]. In an HFD-induced MASLD model, chlorogenic acid (CGA) specifically bound to ALKBH5 and inhibited its m6A demethylase activity [76]. The weakened activity of ALKBH5 reduced the stability of *AXL* mRNA in hepatocytes and downregulated AXL expression, which further suppressed the MAPK/ERK signaling pathway, thus enhancing liver autophagy flux and reducing liver lipid deposition and, finally, improving HFD-induced MASLD [76].

#### 4.1.3. ALKBH5 and T2DM

The preceding results have uncovered a close relationship between ALKBH5 and glucolipid metabolism. However, the role of ALKBH5 in metabolic disorders, especially T2DM, remains controversial (Table 1). Onalan et al. discovered that the expression of FTO and ALKBH5 mRNA in peripheral blood was lower in a T2DM group compared to a healthy group [9]. Shen et al. have reported that the reduced m6A content in the peripheral blood of patients with T2DM and diabetic rats was only related to increased *FTO* mRNA expression, but not to *ALKBH5* [70]. In contrast, Wang et al. revealed that the quantities of Mettl3, Mettl16, and Ythdc2 in the livers of T2DM rats were significantly higher than those in the control group, accompanied by upregulated FTO and Alkbh5 [71]. Thus, it seems that the role of ALKBH5 in T2DM is still controversial and requires further investigation.

### 4.2. ALKBH5 and Immune System Disorders

Immune system function is a crucial self-defense mechanism in humans. Studies have demonstrated that ALKBH5 plays a critical role in a variety of immune system disorders by regulating multiple biological processes, including the development [77] and defense functions [78,79] of immune cells, and the tumor immune microenvironment [56,80] (Table 3).

ALKBH5 may be essential for neutrophil mobilization. Mechanistically, when systemic bacterial infection occurred, ALKBH5 enhanced the expression of pro-neutrophil-migration molecules, such as CXCR2, and promoted the recruitment of neutrophils to the infection area to remove bacteria [78], indicating that ALKBH5 might serve as a key molecule involved in regulating the production of emergency granulocytes. Liu et al. discovered that the high expression of *circZbtb20* reduced the m6A modification level of *Nr4a1* mRNA by enhancing *ALKBH5* expression, thus heightening the stability of *Nr4a1* mRNA [79]. Upregulated *Nr4a1* further activated Notch2 signaling, which was conducive to maintaining the homeostasis of group 3 innate lymphoid cells (ILC3s), thus inhibiting their sensitivity to bacterial infection [79]. This result indicated that ALKBH5 was essential for maintaining ILC3 homeostasis and in the defense against bacterial infection. Unlike the effect on neutrophil antimicrobials, ALKBH5 promoted Salmonella Typhimurium infection by inhibiting the development and maturation of γδT-cell precursor cells in rats [77]. *Alkbh5*-deficient mice exhibited a protective effect against Salmonella typhimurium infection through the downregulation of *Jagged1* and *Notch2* and the promotion of the differentiation and development of γδT-cell precursor cells [77]. The above-mentioned studies revealed that ALKBH5 may function as a marker of bacterial infection. Nevertheless, the conflicting results attributed to the differences in immune cells and bacterial species highlight the complexity of ALKBH5 in bacterial infection immunity.

In addition, ALKBH5 is a major epigenetic regulator of viral infection. Jin and others have reported that *ALKBH5* modulated the expression of *GAS6* through YTHDF2-dependent m6A modification, which attenuated the ability of Porcine Epidemic Diarrhea Virus (PEDV) to infect lung tissue and the 3D4/21 alveolar macrophage cell line [81], indicating that ALKBH5 weakened extra-gastrointestinal PEDV infection. ALKBH5 mediated immune defense against rotavirus (RV) infection as well [82]. Non-structural protein 1 (NSP1) is an RV-encoded innate immune antagonist [87]. Wang et al. discovered that ALKBH5 expression was predominantly diminished in the RV-infected small intestinal epithelial cells (IECs) of mice due to NSP1, which facilitated RV in evading antiviral immune defense [82]. Furthermore, *ALKBH5* enhanced *IFN-I* expression by reducing the m6A modification level of HIV-1 RNA, consequently promoting antiviral immunity in myeloid cells [83]. These findings imply that ALKBH5 might play a principal role in amplifying host resistance in a variety of viral infection-related immune responses.

What is more, ALKBH5 is strongly associated with immune rheumatic diseases. Luo et al. have identified that decreased ALKBH5 expression in peripheral blood was a dangerous factor for rheumatoid arthritis (RA) [84], signaling that ALKBH5 might be involved in the onset of RA. Meanwhile, *ALKBH5* mRNA expression was cardinally cut down in the peripheral blood mononuclear cells of patients with systemic lupus erythematosus (SLE) [85], indicating that ALKBH5 is one of the potential risk factors of SLE [86]. These results suggest that altered ALKBH5 expression levels in peripheral blood can provide novel insights into the pathogenesis of immuno-rheumatic diseases and may serve as a potential biomarker for these diseases.

ALKBH5 has also been demonstrated to shape the tumor immune microenvironment by mediating the M2 polarization of macrophages [56,88] and the immunosuppressive function of bone marrow-derived cells [57,80]. Additionally, ALKBH5 influenced the occurrence and development of various autoimmune diseases, such as autoimmune encephalomyelitis (EAE) [89], autoimmune thyroid disease [90], and primary Sjögren’s syndrome (pSS) [91]. Taken together, it has been established that ALKBH5 is closely associated with bacterial/viral infection and immune system disorders; however, further investigations are required to fully explore the regulatory functions and underlying mechanisms of ALKHB5 in immune diseases.

### 4.3. ALKBH5 and Reproductive System Disorders

The close relationship between ALKBH5 and the occurrence and development of reproductive system disorders, including reproductive system cancers, germ cell development, and abortion, has attracted increasing attention (Table 4).

ALKBH5 promotes the development of reproductive system cancers by increasing the expression of oncogenes. Zhu et al. have reported that *ALKBH5* enhanced the stability of *BCL-2* mRNA in epithelial ovarian cancer, thus enhancing the binding of Bcl-2 and Beclin1, which eventually prohibited autophagy and remarkably increased the proliferation of epithelial ovarian cancer cells [92]. *ALKBH5* raised NANOG expression through the demethylation of *NANOG* mRNA, which accelerated ovarian cancer development [93]. Another research group reported that *ALKBH5* inhibited the degradation of *ITGB1* and strengthened its expression, which augmented the phosphorylation of focal adhesion kinase (FAK) and Src proto-oncogene proteins, and promoted lymph node metastasis [94]. Additionally, the high expression of ALKBH5-mediated demethylation was associated with the metastasis and poor prognosis of various female reproductive system tumors, including cervical cancer [96,97], endometrial cancer [59], and ovarian serous carcinoma [98].

Moreover, ALKBH5 also plays a core role in the growth of germ cells, embracing sperm and oocytes. *Alkbh5* KO mice exhibited upregulated mRNA m6A levels, which affected the output of mRNA, thus suppressing sperm development and sperm quality and ultimately inhibiting the fertility of male mice [25]. The inactivation of ALKBH5 led to male infertility through the promotion of the abnormal splicing of certain transcripts in spermatocyte nuclei [27]. Meanwhile, oocytes with ALKBH5 deficiency exhibited impaired RNA clearance and meiosis disruption, which affected ovarian function and caused female infertility [44]. Generally, m6A modification mediated by ALKBH5 contributes to the development of both spermatocytes and oocytes, suggesting that ALKBH5 may provide novel insights into the potential mechanisms of human infertility.

In addition to its influence on reproductive system cancer progression and germ cell development, ALKBH5 has been reported to impact female miscarriage as well. Li et al. demonstrated that the absence of *ALKBH5* in patients with recurrent miscarriage (RM) obstructed trophoblast invasion by extending the half-life of *CYR61* mRNA [44]. Alternatively, Zheng et al. reported that *ALKBH5* reduced the risk of spontaneous miscarriage by increasing the activity of trophoblasts through the upregulation of SMAD1/5 by removing m6A methylation in *SMAD1/5* mRNA [95].

In conclusion, the above-mentioned studies suggest that ALKBH5 is tightly associated with reproductive system diseases, and it may serve as a potential therapeutic target for reproductive system tumors, germ cell development, and abortion, providing new strategies for therapeutically treating reproductive system diseases.

### 4.4. ALKBH5 and Nervous System Disorders

Research has shown that ALKBH5 expression undergoes significant changes in the biological processes of various neurological diseases (Table 5). Du et al. discovered that ALKBH5 was clearly decreased during brain growth [99]. Wang et al. revealed that the knockdown of ALKBH5 not only promoted sensory axon regeneration in the peripheral and central nervous systems, but also enhanced the survival of retinal ganglion cells following optic nerve damage [100], indicating the important role of ALKBH5 in the survival and function of neurons. Moreover, Meng et al. unveiled that in hippocampal neuronal injury mice, Alkbh5 expression was increased in the hippocampi, accompanied by learning and memory impairments. And IOX1 treatment, an ALKBH5 inhibitor, significantly improved learning and memory defects [101], which further indicated the critical role of ALKBH5 in regulating neural networks. On the contrary, Xu et al. reported that the global RNA m6A level was increased in the brain tissue of middle cerebral artery occlusion rats, and the inhibition of *Alkbh5* promoted OGD/R-induced neuronal damage [102]. Previous studies suggest that regulating the expression and catalyzed activity of ALKBH5 may offer a new strategy for interventions in neurodevelopmental disorders.

In addition, ALKBH5 has been considered to be involved in the pathogenesis of mental disorders, such as depression and anxiety (Table 5). Guo et al. reported that the upward expression of *ALKBH5* in astrocytes lowered *GLT-1* m6A modification under stress conditions, which impaired glutamic acid intake, thereby promoting depressive-like symptoms [51]. Another study has shown that upregulated *ALKBH5* demethylated *Htr3a* mRNA and increased the expression of the 5-HT3A protein and channel current, which promoted neuropathological pain mediated by the trigeminal nerve [103]. These findings reveal the vital role of ALKBH5 in mental illness and highlight its potential as a treatment target for neuropathy.

In summary, these studies unveil the important regulatory role of ALKBH5 in neurodevelopment and neurodegenerative and mental illness.

## 5. Development and Potential Applications of ALKBH5 Inhibitors

Since the critical role of ALKBH5 in various diseases has been discovered, the development of inhibitors targeting ALKBH5 has attracted more attention. And recently, several ALKBH5 inhibitors with promising applications have been identified, including natural, clinical, pharmacological, and small-molecule inhibitors (Table 6).

Currently, the main natural ALKBH5 inhibitors are citrate and chlorogenic acid (CGA). Xu et al. identified that citrate replaced metal ions and 2-oxoglutarate (2OG) by directly binding to ALKBH5 and then disrupting the demethylase activity of ALKBH5, thus naturally inhibiting ALKBH5 [104]. In addition, CGA has been reported to enhance autophagy and improve liver fat degeneration by inhibiting ALKBH5 activity [76].

A variety of clinical pharmacological inhibitors of ALKBH5 have been developed. It was reported that IOX1, a broad-spectrum inhibitor of 2-OG oxygenases, suppressed ALKBH5 expression by competing with 2-OG [105], which prevented acute kidney injury (AKI) [106] and age-related macular degeneration (AMD) [107]. Furthermore, Dexmedetomidine, an α2-adrenenergic receptor agonist, has been discovered to inhibit ALKBH5 activity, which provided a novel approach for preventing and treating septic kidney injury [108]. And ALK-04, a compound synthesized by in silico screening using the X-ray crystal structure of ALKBH5, was identified as a specific ALKBH5 inhibitor that improved the efficacy of cancer immunotherapy for patients with melanoma [109]. Another drug, known as 20m, which was obtained through fluorescence-polarization-based screening, structural optimization, and structure–activity relationship analysis, has also proved to be a potent, selective, and cell-active inhibitor of ALKBH5 [110], effectively inhibiting the expression of ALKBH5 and exhibiting a protective effect in oxygen glucose deprivation (OGD)-induced brain microvascular endothelial cell (BMEC) injury [111].

In addition, several new small-molecule inhibitors of ALKBH5 have been developed. For example, Ena15 is considered a non-competitive inhibitor of ALKBH5, while Ena21 is a competitive inhibitor of ALKBH5; both have been demonstrated to suppress the progression of glioblastoma [112]. A pyrazolo and [1,5-a] pyrimidine derivative (DO-2728) increased the m6A level in AML cells by specifically downregulating ALKBH5 [113], which eventually inhibited tumor growth. Compounds such as 2-[(1-hydroxy-2-oxo-2phenylethyl) sulfanyl] acetic acid (3) (cmp-3) and 4-{[(furan-2-yl)-methyl] amino}-1,2-diazinane-3,6-dione (6) (cmp-6) have been identified as ALKBH5 inhibitors as well, significantly suppressing cancer progression [114]. The covalent inhibitor TD19 prevented ALKBH5 from binding to RNA m6A-methylated sites, thereby exerting an anti-cancer effect [115]. Additionally, imidazobenzoxazine-5-thione (MV1035) inhibited the demethylation activity of ALKBH5 by competing with the 2OG active site, ultimately suppressing the migration and invasion of GBM cells [116,117].

All in all, the above-mentioned inhibitors effectively decrease the activity of ALKBH5, thus affecting the m6A level in target mRNAs, which provides a novel therapeutic strategy for various human diseases, especially in cancer treatment. However, further investigations are required to identify more inhibitors and unveil their effects and underlying mechanisms for specific diseases.

## 6. Conclusions and Perspectives

As one of the two major demethylases for dynamic and reversible m6A methylation, ALKBH5 has been established to be involved in diverse physiological and pathological processes through regulating the expression of numerous genes at the post-transcriptional level [27,28,29,30,31,32,33,34,35] and maintaining the balance between RNA methylation and demethylation.

Extensive studies have revealed the dual role of ALKBH5 in multiple biological processes. ALKBH5 regulates the m6A level of target genes to interdict carcinogenesis by suppressing glycolysis in bladder cancer [66], prostate adenocarcinoma (PARD) [67], and colorectal cancer [68], while in drug-resistant breast cancer, ALKBH5 promotes cancer progression by enhancing glycolysis [33], indicating a dual role of ALKBH5 in regulating glycolysis which warrants further investigation. In addition, whether ALKBH5 promotes adipogenic differentiation [74] or inhibits adipogenesis [72,73] may depend on different cell types or species. The same can be said for the complexity of the m6A mechanism in bacterial infection immunity; ALKBH5 may be a potential treatment target for bacterial infection [78,79], or may act as a risk factor for bacterial infection according to the type of bacterial infection and immune cells [77]. Thus, the effect of ALKBH5 on a multitude of physiological and pathological processes is still ambiguous and requires further investigation. Dealing with these problems requires precise individualized diagnoses and treatments.

Previous studies have shown that ALKBH5 has a dual role in several diseases, which may be due to different cell types, tissues, or species, thus leading to limitations in the application of ALKBH5 inhibitors in these diseases. Nonetheless, ALKBH5 and its inhibitors have considerable clinical value in other diseases, especially in cancer. For example, research shows that during the occurrence and progression of GBM, *ALKBH5* mainly acts as an oncogene; using ALKBH5 inhibitors, such as Ena15 [112], Ena21 [112], cmp3 and cmp6 [114], or MV1035 [116,117], effectively inhibits the progression of GBM. And DO-2728 [113] or TD19 [115] suppresses the progression of AML through inhibiting ALKBH5. In addition, the use of the ALKBH5 inhibitor IOX1 significantly improves the progression of AKI [106] and AMD [107]. Generally, ALKBH5 inhibitors have a wide range of potential applications in some diseases, and more inhibitors are expected to be developed and applied in clinical treatment in the future.

In this review, we comprehensively summarized the structure and biological functions of AKBH5, and the critical roles of ALKBH5 in various diseases, as well as the development and application of ALKBH5 inhibitors, not only helping to deepen the understanding of the complexity of the regulatory mechanisms of m6A modification in human diseases, but also providing novel insights to support prognoses and therapies for related diseases. However, further studies are still required to probe the precise effects and detailed molecular mechanisms of ALKBH5 in human diseases to ultimately promote the use of ALKBH5 inhibitors for clinical applications, and especially to fully elucidate the dual role of ALKBH5 in several biological processes.

## Figures and Tables

**Table 1:** Expression and clinical significance of ALKBH5 in glucose metabolism.

Glucose Metabolism	ALKBH5	Target	Function	References
Enhanced Glycolysis	down	*CK2α*	Downregulated *ALKBH5* promoted bladder cancer development through modulating the glycolysis pathway mediated by *CK2α* in an m6A-dependent manner.	[66]
	down	*FLII*	The USF1-mediated downregulation of *ALKBH5* stabilized *FLII* mRNA in a YTHDF2-dependent manner to repress glycolytic activity, subsequently inhibiting prostate adenocarcinoma.	[67]
	down	*HK2*	In a high-fat environment, the diminished expression of *FTO* and *ALKBH5* cooperatively activated FOXO signaling through IGF2BP2-mediated m6A methylation in *HK2* mRNA, which boosted glycolysis in colorectal cancer.	[68]
	up	*GLUT4*	The increased expression of *ALKBH5* promoted the m6A demethylation and stability of *GLUT4* mRNA in a YTHDF2-dependent manner, leading to enhanced glycolysis in drug-resistant breast cancer cells.	[33]
Aerobic Glycolysis	up	*LDHA*	*PRMT6* directly methylated ALKBH5 at Arg283, which strengthened the stability of *LDHA* mRNA, leading to increased aerobic glycolysis in breast cancer cells.	[60]
Pentose Phosphate Pathway (PPP)	up	*G6PD*	Upregulated *ALKBH5* demethylated *G6PD* mRNA and enhanced the stability and expression of G6PD, which activated the pentose phosphate pathway and stimulated the proliferation of glioma cells.	[69]
T2DM (Type 2 Diabetes Mellitus)	down	-	The expression of *FTO* and *ALKBH5* mRNA in peripheral blood was lower in the T2DM group compared to the healthy group.	[9]
	unchanged	-	The reduced m6A content in the peripheral blood of patients with T2DM and diabetic rats was only related to increased *FTO* mRNA expression, but not *ALKBH5*.	[70]
	up	-	FTO and Alkbh5 quantities in the liver of T2DM rats were higher than those in the control group.	[71]

**Table 2:** Expression and clinical significance of ALKBH5 in lipid metabolism.

Lipid Metabolism	ALKBH5	Target	Function	References
Adipogenesis	down	*LCAT*	Low expression of ALKBH5 reinforced the m6A methylation of *LCAT* to improve the stability of its mRNA, which promoted preadipocyte differentiation and thus enhanced adipogenesis.	[72]
	down	*TRAF4*	Downregulated ALKBH5 enhanced TRAF4 m6A modification, thus reducing the expression of TRAF4, and the PKM2/TRAF4 interaction, which weakened the kinase activity of PKM2 and obstructed β-catenin signal transduction, thus promoting the fat formation of MSCs.	[73]
	up	*TRAF4*	Curcumin reduced the expression of ALKHB5, leading to an increase in m6A-modified *TRAF4* mRNA and promoting its translation, which promoted the degradation of adipocyte differentiation regulator PPARγ through a ubiquitin–proteasome pathway, thereby inhibiting adipogenesis.	[74]
Lipid metabolism	up	*FABP5*	Upregulated *ALKBH5* significantly increased *FABP5* expression in an m6A-IGF2BP2-dependent manner, thereby activating the PI3K/Akt/mTOR signaling pathway and enhancing lipid metabolism in pNENs.	[75]
Lipid deposition	up	*AXL*	The weakened activity of ALKBH5 mediated by CGA reduced the stability and expression of *AXL* mRNA in hepatocytes, which further suppressed the MAPK/ERK signaling pathway, thus reducing liver lipid deposition and, finally, improving HFD-induced MASLD.	[76]

**Table 3:** Expression and clinical significance of ALKBH5 in immune diseases.

Disease	ALKBH5	Target	Function	References
Systemic bacterial infection	down	*CSF3R*	When systemic bacterial infection occurred, ALKBH5 enhanced the expression of pro-neutrophil-migration molecules such as CXCR2, thereby promoting the recruitment of neutrophils to the infection area to remove bacteria.	[78]
*C. rodentium* infection	down	*Nr4a1*	High expression of *Alkbh5* reduced the m6A level of *Nr4a1* mRNA and heightened its stability, which activated Notch2 signaling, maintaining the homeostasis of group 3 innate lymphocyte cells (ILC3s), thereby reducing susceptibility to *C. rodentium* infection.	[79]
Gastrointestinal *Salmonella typhimurium* infection	up	*Jagged1 and Notch2*	*Alkbh5*-deficient mice exhibited a protective effect against *Salmonella typhimurium* infection through the downregulation of *Jagged1* and *Notch2*.	[77]
PEDV infection	down	*GAS6*	ALKBH5 modulated the expression of *GAS6*, which attenuated the ability of PEDV to infect lung tissue and the 3D4/21 alveolar macrophage cell line.	[81]
RV infection	down	*NSP1*	ALKBH5 expression was predominantly diminished in the RV-infected IECs of mice due to NSP1, which facilitated the RV virus in evading antiviral immune defense.	[82]
HIV-1 infection	down	*IFN-I*	*ALKBH5* reduced the m6A level of HIV-1 RNA to enhance the expression of *IFN-I* by activating transcription factors IRF3 and IRF7, thus promoting the antiviral immunity of bone marrow cells.	[83]
RA	down	-	A decreased peripheral blood expression of ALKBH5 was a dangerous factor for rheumatoid arthritis.	[84]
SLE	down	-	*ALKBH5* mRNA expression was cardinally cut down in the peripheral blood mononuclear cells of patients with SLE, implicating ALKBH5 as one of the potential risk factors of SLE.	[85,86]

**Table 4:** Expression and clinical significance of ALKBH5 in reproductive diseases.

Disorder	ALKBH5	Target	Function	References
Epithelial ovarian cancer	up	*BCL-2*	ALKBH5 promoted the stability of *BCL-2* mRNA and thus enhanced the binding of Bcl-2 and Beclin1, which eventually prohibited autophagy and aggravated epithelial ovarian cancer.	[92]
Ovarian cancer	up	*NANOG*	*ALKBH5* enhanced NANOG expression through the demethylation of *NANOG* mRNA, which accelerated ovarian cancer development.	[93]
Metastatic ovarian cancer	up	*ITGB1*	*ALKBH5* inhibited the degradation of *ITGB1* and enhanced its expression, which augmented the phosphorylation of focal adhesion kinase (FAK) and Src proto-oncogene proteins, and promoted lymph node metastasis.	[94]
Endometrial cancer	up	*IGF1R*	*ALKBH5* promoted the proliferation and invasion of endometrial cancer via the erasing of *IGF1R* m6A modifications.	[59]
Infertility	down	-	*ALKBH5* KO in mice affected the output of mRNA and thus suppressed sperm development and quality, ultimately inhibiting fertility.	[25]
	down	*Unc50* and *Traf3ip1*	The inactivation of Alkbh5 in spermatocytes and round sperm nuclei led to abnormal splicing and the production of shorter transcripts, resulting in male infertility in mice.	[27]
	down	*Atp5j2*, *Birc5*, *Esrrb*, and *Rpl39*	The loss of *Alkbh5* caused oocyte meiosis defects, leading to impaired RNA clearance and female infertility.	[45]
Recurrent miscarriage (RM)	up	*CYR61*	In the trophoblast of patients with RM, upregulated *ALKBH5* shortened the half-life of *CYR61* mRNA and inhibited its expression, thereby inhibiting trophoblast invasion.	[44]
Recurrent spontaneous abortion (RSA)	down	*SMAD1 / 5*	The trophoblast-specific knockdown of *ALKBH5* in mouse placenta attenuated the translation of *SMAD1/5* by increasing m6A modification, thereby inhibiting trophoblast cell activity and significantly leading to fetal abortion.	[95]

**Table 5:** Expression and clinical significance of ALKBH5 in neurological processes or diseases.

Process or Disease	ALKBH5	Target	Function	References
Brain development	down	-	Alkbh5 protein decreased dramatically during brain development.	[99]
Optic nerve injury	up	*Lpin2*	*ALKBH5* increased the stability of *Lpin2* mRNA and thus hindered the regenerative growth associated with lipid metabolism in neurons, thereby inhibiting survival and axonal regeneration after neuronal injury in rodents.	[100]
Learning and memory impairments	up	-	In hippocampal neuronal injury mice, Alkbh5 expression was increased in the hippocampus, accompanied by learning and memory impairments.	[101]
Cerebral I/R injury	down	*SNHG3*	*ALKBH5* induced *SNHG3* mRNA demethylation to inhibit its expression, thereby protecting against damage and PANoptosis in a cerebral I/R injury model.	[102]
Major depression disorder (MDD)	up	*GLT-1*	*ALKBH5* lowered *GLT-1* m6A modification and increased the expression of *GLT-1* in astrocytes, thereby impairing glutamate uptake and, finally, promoting depressive-like behaviors.	[51]
Neuropathic pain	up	*Htr3a*	The *FOXD3*-mediated transactivation of *ALKBH5* promoted neuropathic pain through the m6A-dependent stabilization of *Htr3a* mRNA in trigeminal ganglion (TG) neurons.	[103]

**Table 6:** Development and application of ALKBH5 inhibitors.

Inhibitor	Type	Selectivity	Diseases	References
Citrate	Natural inhibitor	-	-	[104]
CGA	Natural inhibitor	No	MASLD	[76]
IOX1	Competitive inhibitor	No	I/R-induced renal injury	[105]
			AKI	[106]
			AMD	[107]
Dexmedetomidine	Demethylase activity inhibitor	No	Sepsis	[108]
ALK-04	Small-molecule inhibitor	No	Melanoma	[109]
20m	Novel inhibitor	Yes	OGD-induced BMEC injury	[110,111]
Ena21	Competitive inhibitor	No	GBM	[112]
Ena15	Non-competitive inhibitor	Yes	GBM	[112]
DO-2728	Competitive inhibitor	Yes	AML	[113]
cmp-3 and cmp-6	Novel inhibitor	Yes	Leukemia and GBM	[114]
TD19	Covalent inhibitor	Yes	AML	[115]
MV1035	Competitive inhibitor	Yes	GBM	[116,117]

## Data Availability

Not applicable.
